# Effect of Omega-3 Supplementation in Pregnant Women with Obesity on Newborn Body Composition, Growth and Length of Gestation: A Randomized Controlled Pilot Study

**DOI:** 10.3390/nu13020578

**Published:** 2021-02-09

**Authors:** Carmen Monthé-Drèze, Sarbattama Sen, Sylvie Hauguel-de Mouzon, Patrick M. Catalano

**Affiliations:** 1 Department of Pediatric Newborn Medicine, Brigham and Women’s Hospital, Boston, MA 02115, USA; ssen2@bwh.harvard.edu; 2 School of Medicine, Harvard University, Boston, MA 02115, USA; 3 Department of Reproductive Biology, Case Western Reserve University, Cleveland, OH 44106, USA; sxh120@case.edu; 4 Mother Infant Research Institute, Tufts Medical Center, Boston, MA 02111, USA; pcatalano@tuftsmedicalcenter.org

**Keywords:** omega-3 supplementation, fish oil, pregnancy, obesity, gestational age, birthweight, fetal growth, body composition, newborn fat mass, newborn lean mass

## Abstract

Maternal obesity, a state of chronic low-grade metabolic inflammation, is a growing health burden associated with offspring adiposity, abnormal fetal growth and prematurity, which are all linked to adverse offspring cardiometabolic health. Higher intake of anti-inflammatory omega-3 (n-3) polyunsaturated fatty acids (PUFA) in pregnancy has been associated with lower adiposity, higher birthweight and longer gestation. However, the effects of n-3 supplementation specifically in pregnant women with overweight and obesity (OWOB) have not been explored. We conducted a pilot double-blind randomized controlled trial of 72 pregnant women with first trimester body mass index (BMI) ≥ 25 kg/m^2^ to explore preliminary efficacy of n-3 supplementation. Participants were randomized to daily DHA plus EPA (2 g/d) or placebo (wheat germ oil) from 10–16 weeks gestation to delivery. Neonatal body composition, fetal growth and length of gestation were assessed. For the 48 dyads with outcome data, median (IQR) maternal BMI was 30.2 (28.2, 35.4) kg/m^2^. In sex-adjusted analyses, n-3 supplementation was associated with higher neonatal fat-free mass (β: 218 g; 95% CI 49, 387) but not with % body fat or fat mass. Birthweight for gestational age z-score (−0.17 ± 0.67 vs. −0.61 ± 0.61 SD unit, *p* = 0.02) was higher, and gestation longer (40 (38.5, 40.1) vs. 39 (38, 39.4) weeks, *p* = 0.02), in the treatment vs. placebo group. Supplementation with n-3 PUFA in women with OWOB led to higher lean mass accrual at birth as well as improved fetal growth and longer gestation. Larger well-powered trials of n-3 PUFA supplementation specifically in pregnant women with OWOB should be conducted to confirm these findings and explore the long-term impact on offspring obesity and cardiometabolic health.

## 1. Introduction

The prevalence of pre-pregnancy overweight and obesity (OWOB) is increasing worldwide [1] and is currently over 60% in the United States [2,3]. Pre-pregnancy obesity is associated with higher adiposity at birth, abnormal fetal growth (large and small for gestational age infants) and medically indicated and spontaneous preterm births, which together are amongst the strongest pre- and perinatal risk factors for childhood obesity and adverse cardiometabolic health later in life [4,5,6,7,8,9].

Maternal obesity-associated inflammation has been found to be associated with altered placental lipid metabolism and insulin signaling that are thought to lead to excessive nutrient transport, resulting in increased fetal fat accretion [10,11]. On the other hand, placental inflammation and lipotoxicity may also result in abnormal placentation and vasculature that may lead to placental insufficiency, resulting in intra-uterine growth restriction and preterm births [11]. Thus, there can be marked heterogeneity in fetal growth patterns in pregnant women with OWOB. This adverse in-utero metabolic milieu is associated with metabolic programming in the offspring with consequences throughout the lifespan [6,11,12].

Polyunsaturated fatty acids (PUFA) can modulate inflammation, insulin sensitivity and lipid metabolism, and the PUFA profile during pregnancy has been associated with length of gestation and offspring adiposity [10]. A recently published Cochrane review showed that n (omega)-3 PUFA supplementation was associated with reduced risk of preterm birth and low birthweight (BW) [13]. Prospective cohort studies have shown associations of higher n-6 PUFA with lower birthweight (BW), higher fat mass (FM) and higher body fat, and associations of higher n-3 with higher lean mass (LM) and lower adiposity in childhood [14,15,16]. Animal studies have shown that interventions to increase the anti-inflammatory omega-3 (n-3) to pro-inflammatory n-6 ratio can reduce obesity-induced inflammation and insulin resistance and prevent offspring adverse metabolic programming [17,18]. Despite these findings, several n-3 randomized controlled trials (RCTs) have reported mixed findings [19,20,21,22,23], and systematic reviews have not been conclusive on the effects of n-3 supplementation on neonatal outcomes, specifically offspring body composition [24,25,26]. Methodological limitations may explain these inconsistencies, including participant characteristics, timing of initiation of intervention, duration of supplementation, and dose and composition of supplements. 

Animal studies [10,12,17,18] and post-hoc analyses from prior RCTs [20,23,27,28,29] suggest the biological plausibility that women with higher baseline metabolic dysregulation and with low n-3 status may most benefit from dietary supplementation strategies. However, studies evaluating the role of n-3 PUFA supplementation specifically in these higher risk cohorts are lacking. We and others have shown that women with obesity have lower n-3 and higher n-6 concentrations compared with normal-weight mothers [30,31] and are more likely to consume a more pro-inflammatory “Western diet” [32,33], further perturbating the in-utero fetal milieu. We also previously reported that in women with OWOB only, higher n-6/n-3 PUFA ratio was associated with shorter length of gestation and impaired fetal growth [34]. However, the effects of n-3 PUFA supplementation on length of gestation and neonatal outcomes, specifically in women with OWOB, are still unknown.

We conducted a pilot double-blind RCT in healthy women with body mass index (BMI) > 25 kg/m^2^ to investigate whether n-3 supplementation starting in early gestation (<16 weeks) would improve maternal insulin sensitivity and reduce inflammation, the co-primary outcomes of this trial. We have previously reported on these outcomes and shown that n-3 PUFA supplementation reduced systemic and placental inflammation, though there was no significant effect on insulin resistance [35]. We have also reported, in a secondary analysis of the trial, that n-3 PUFA supplementation reduced placental lipid storage [36]. 

The objective of the present study was to explore the preliminary efficacy of n-3 PUFA supplementation on neonatal adiposity (primary outcome), and fetal growth and length of gestation (secondary outcomes). Neonatal adiposity was a secondary outcome measure in this pilot RCT, though this measure will be evaluated as the primary outcome for this manuscript. We aimed to explore preliminary evidence of efficacy and help inform future larger well-powered trials of n-3 supplementation in pregnant women with overweight and obesity. Fetal growth and length were not specified as outcomes in the original study design and thus are secondary analyses of the data. Therefore, the present study is not powered to assess the effects of n-3 PUFA supplementation on these outcomes. Given their anti-inflammatory and insulin-sensitizing properties, we hypothesized that n-3 PUFA supplementation in women with OWOB would result in lower adiposity, higher fetal growth, and longer gestation. This study was supported by an American Recovery and Reinvestment Act (ARRA) 2-year grant from the National Institutes of Health RHD057236.

## 2. Methods

### 2.1. Study Design and Intervention

**Figure 1:**
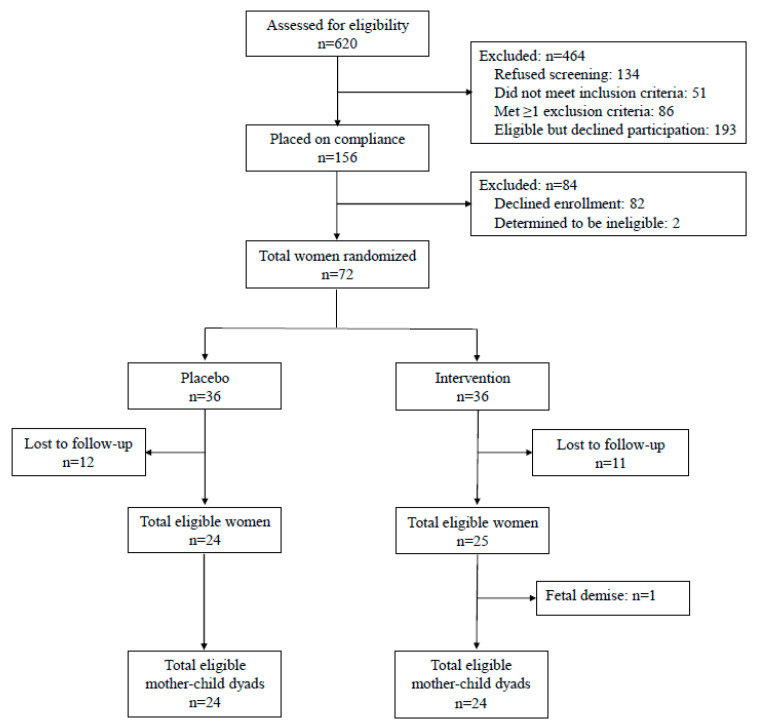
Flow diagram of the included participants. n: number.

We conducted a pilot double-blind RCT in healthy women with BMI > 25 kg/m^2^ at MetroHealth Medical Center/Case Western Reserve University. Participants were randomized in early gestation (<16 weeks) to either oral n-3 supplements containing 800 mg docosahexaenoic acid (DHA) and 1200 mg eicosapentaenoic acid (EPA) for a total of 2000 mg of n-3 PUFA, divided into four capsules, or matching placebo capsules contained wheat germ oil. All subjects were instructed to take two capsules twice a day from enrollment until delivery. Details of the study design, inclusion and exclusion criteria and methods of the trial were described previously [35]. Briefly, inclusion criteria were a confirmed singleton pregnancy and BMI > 25 kg/m^2^ at the first antenatal visit without evidence of existing metabolic disorder such as hypertension, diabetes or hyperthyroidism. Of the 620 women who were screened, 156 agreed to be enrolled in the one-week run-in, and of these women, 84 failed the run-in as they did not return or had taken less than 50% of the placebo capsules. The 72 participants who passed the compliance run-in were randomized using a computer-generated randomization table (generated by Eminent Corporation, Frederick, MD, USA, the supplier of the n-3 capsules and placebo). Women were allocated to study arms by Eminent according to the randomization list and we received identical packets with unique study numbers for each participant. Eminent Corporation had no contact with participants and had no role in either study recruitment or its execution. Of the 72 women who passed the run-in, 23 were lost to follow-up due to participant burden in light of the various metabolic studies which were part of the protocol. Forty-nine women completed the trial. For the current analysis, we additionally excluded one mother-child dyad due to fetal demise. We included n = 48 mother-infant dyads in this analysis (24 placebo and 24 supplemented, Figure 1). Study group assignment was blinded and not known by study participants, their health care providers, or study personnel and assessors. The codes of n-3 and placebo were broken only after completion of the study. Blister packs were dispensed monthly at routine obstetrical visits at which time compliance (pill counting) and side effect were assessed. There were two visits in the clinical research unit (CRU) at MetroHealth Medical Center for each study participant: visit one (V1) between 8–16 weeks and visit 2 (V2) between 34–36 weeks. Procedures that are specific to the current analysis are described below.

### 2.2. Ethical Approval 

This study was conducted according to the guidelines of the Declaration of Helsinki and all procedures were approved by the Institutional Review Board of MetroHealth Medical Center/Case Western Reserve University and Brigham and Women’s Hospital. Written informed consent was obtained from all participants at study entry. The project was registered at clinicaltrials.gov as NCT00957476. 

### 2.3. Maternal Measures

#### 2.3.1. Baseline Characteristics

Maternal demographic, medical and pregnancy history data were collected by trained research assistants via interviews and questionnaires. Maternal height and weight were collected at V1 and V2. Maternal height was measured with a stadiometer to the nearest 1.0 mm and weight was measured with a calibrated scale (Mettler Toledo, Inc., Toledo, OH, USA) to the nearest 0.01 kg. BMI was calculated as kg/m^2^ and categories were defined per the WHO criteria: Overweight ≥25 and <30 kg/m^2^ and obese ≥30 kg/m^2^. Gestational weight gain was calculated by subtracting weight at V1 from V2.

#### 2.3.2. Maternal Dietary Assessment

At the first and second visits, participants completed a semiquantitative food-frequency questionnaire (FFQ) which was modified for use in pregnancy from a well-validated instrument used in several large cohorts of non-pregnant adults [37], and validated against plasma biomarkers in pregnant women [38,39]. The FFQ quantified the average frequency consumption of >140 specific foods. We used the Harvard nutrient composition database, which is based primarily on USDA publications, to obtain estimates of nutrients. At V1, the FFQ assessed diet intake over the prior year; at V2, it assessed diet intake since the baseline visit, reflecting dietary intake during the second and third trimesters.

#### 2.3.3. Fatty Acid Plasma Assays

Maternal blood was collected at V1 and V2. Trained research staff at the CRU immediately separated the plasma from erythrocytes and stored aliquots in liquid nitrogen. Details of the fatty acids (FA) analysis were described previously [35]. Briefly, total lipids were extracted from maternal plasma with 2:1 (*v*/*v*) chloroform:methanol and washed with 0.88% KCl per the method described by Foch et al. [40] Fatty acid methyl esters were prepared using 5% HCl in methanol at 76 °C. Analysis of fatty acid methyl esters was completed by gas chromatography. Retention times were compared to standards (Matreya, LLC, Pleasant Gap, PA, Supelco, Bellefonte, PA, and Nu-Check Prep Inc., Elysian, MN, USA) and FA are reported as percent molar of total identified.

### 2.4. Neonatal Measures 

#### 2.4.1. Neonatal Body Composition

We assessed body composition two ways: (1) we estimated neonatal FM from BW, length, and flank skin fold thickness (SFT) using a previously validated anthropometric equation [41]. Fat free mass (FFM) was calculated as BW-FM. Body fat percentage (BF%) was calculated as 100 × FM/BW (2). We also assessed body composition during the first 72 h of life by air displacement plethysmography (ADP) using the PeaPod^TM^ Body Composition System (COSMED, Rome, Italy). The PeaPod was administered twice for each infant. If the percent of body fat differed by >0.2%, the test was repeated a third time. For each outcome, the average of the two closest measures was used. Using pressure-volume equations, FM and FFM were estimated to provide BF% which was derived as 100 × FM/(FM + FFM).

#### 2.4.2. Neonatal Anthropometry and Fetal Growth

Neonatal anthropometric measurements, including BW, length, and flank SFT, were obtained within 72 h of delivery. Detailed methods for neonatal anthropometric measurements have been described previously [42]. Fetal growth was assessed using sex-specific BW for gestational age *z* score (BWGA-z) and birth length for gestational age *z*-score (BLGA-z). All *z* scores were calculated using the Fenton 2013 clinical calculator [43]. Also using the Fenton standards, we estimated growth percentiles and categorized babies as small for gestational age (SGA) if BW < 10th %ile, and large for gestational age (LGA) if BW > 90th %ile.

### 2.5. Length of Gestation

Length of gestation was calculated based on last menstrual period and confirmed based on dating ultrasound.

### 2.6. Statistical Analyses

All statistical analyses were performed using Stata/SE version 15.1 (StataCorp., College Station, TX, USA). A two-sided *p*-value < 0.05 was considered statistically significant for all analyses. Data were analyzed using intention-to-treat. Descriptive statistics were used to characterize maternal and neonatal characteristics by treatment group after testing for normal distribution. We used the Wilcoxon-rank sum test to compare dietary intake and FA concentrations between treatment groups. We used the Wilcoxon signed-rank tests to determine if dietary intake and FA concentrations following supplementation were significantly different from baseline within each treatment group. We assessed Pearson or Spearman’s correlations between Peapod and anthropometric measures of body composition. We used the Student *t* test or Wilcoxon-rank sum test for continuous variables, and the *χ*^2^ test for categorical variables, for comparisons of outcome measures between treatment groups. We then performed multivariable linear regression analyses to estimate differences between treatment groups (placebo as reference) in measures of body composition adjusted for sex since neonatal body composition differ in males vs. females. We did not adjust for maternal characteristics since this was a RCT and baseline characteristics were similar between groups. Finally, we use linear regression analyses in stratified analyses to determine whether estimates of the difference between treatment groups (placebo as reference) in the outcome measures differ by first trimester BMI category (overweight vs. obese), maternal n-6/n-3 baseline dietary intake (below vs. above median intake), and by offspring sex. Prior studies have shown that n-3 supplementation may be beneficial specifically in women with overall lower dietary quality (low dietary and plasma n-3 PUFA) and with the highest metabolic dysfunction. Furthermore, animal data have shown sexual dimorphism in the effects of maternal dysmetabolism, inflammation and overnutrition on fetal development with males being more susceptible to the in-utero environment. Therefore, we hypothesized that male offspring as well as women with obesity and with the highest dietary n-6/n-3 intake, a marker of dietary inflammation, would benefit most from n-3 supplementation.

## 3. Results

### 3.1. Participant Characteristics

The baseline characteristics of the participants are summarized in Table 1 Compared to the women who failed run-in, the women who were randomized were older (mean ± SD: 26.9 ± 0.6 years vs. 25.1 ± 0.6 years; *p* = 0.03) and more likely to be White (42% vs. 27%, *p* = 0.045). 

Compared to the women who were lost to follow-up, the women who completed the study had lower baseline BMI (median (IQR): 30 (28, 35) kg/m^2^ vs. 33 (31, 42) kg/m^2^; *p* = 0.04) and were more likely to be nulliparous (67% vs. 33%; *p* < 0.001). Among the 48 included participants, the mean ± SD maternal age at enrollment was 26.9 ± 5 years. The median and interquartile range (IQR) maternal BMI and gestational age (GA) at recruitment were 30.2 (28.2, 35.4) kg/m^2^ and 14.8 (13, 15.6) weeks. Amongst all the women, 44% were white, 35% were African American, 50% were overweight and 50% had obesity. There were no differences in maternal characteristics between placebo and treatment groups. Five participants were diagnosed with gestational diabetes melitus (GDM) and were diet-controlled. Among the children, 52% were female and 48% were male.

### 3.2. Maternal Dietary Intake

In Table 2, we report the median (IQR) maternal intake for macronutrient and FA at V1 and V2 by treatment group. There were no significant differences in calories, macronutrients, n-3 and n-6 PUFA, n-6/n-3 PUFA ratio, total PUFA and saturated fatty acids (SFA) intake between the placebo and treatment groups at either time point (V1 and V2) during pregnancy. Similarly, there were no differences in the intake of these nutrients during pregnancy compared to pre-pregnancy within each group (all *p* > 0.05).

### 3.3. Maternal Plasma Fatty Acids Concentrations

Plasma FA concentrations at V1 and V2 by treatment group are presented in Table 3. At baseline (V1), there were no differences between n-3 vs. placebo groups in the plasma concentrations of EPA, DHA, DHA+EPA, total n-3, total n-6, AA, n-6/n-3 ratio, AA/DHA+EPA ratio and SFA. At 34–36 weeks (V2), the plasma concentrations of EPA DHA, DHA+EPA and total n-3 were higher in the n-3 vs. placebo group (all *p* < 0.05). The ratios of total n-6/n-3 and of AA/DHA+EPA were lower in the n-3 vs. placebo group at V2 (all *p* < 0.05).

### 3.4. Neonatal Body Composition 

FM (r = 0.67, *p* < 0.001), FFM (r = 0.93, *p* < 0.001), and BF% (r = 0.38, *p* = 0.01) measures from the validated anthropometric method and PeaPod method were highly correlated. Results using the anthropometric method were available for all 48 newborns and presented in Table 4 In sex-adjusted analyses, newborns of mothers supplemented with n-3 PUFA had higher FFM (β 218 g, 95% CI: 49, 387) compared to newborns of mothers in the placebo group (Table 4); this association was still significant after additional adjustment for gestational age (β 126 g, 95% CI: 3, 249).

In unadjusted analyses, newborns of mothers supplemented with n-3 PUFA had higher FM. However, in sex-adjusted analyses, results were attenuated to the null (Table 4). 

Similarly, %BF did not differ between the n-3 PUFA vs. placebo groups (β 1.2 %, CI −1, 3.4) (Table 4) in adjusted analyses. Results for PeaPod-derived body composition measures are summarized in Table S1 for the 40 newborns with available PeaPod data. FFM estimated using PeaPod was significantly higher in the n-3 PUFA vs. placebo group, though the confidence interval included the null (β 182 g, CI: −4, 369) after adjustment of newborn sex. Similar to the validated anthropometry-derived body composition measures, there were no differences between the two groups in FM and % body fat in adjusted analyses. 

### 3.5. Birthweight and Fetal Growth

Newborns of mothers supplemented with n-3 PUFA during pregnancy had higher BW (mean ± SD n-3: 3278 ± 448 g; placebo: 2935 ± 356 g) and BWGA-z (n-3: −0.17 ± 0.67 SD units vs. placebo: −0.61 ± 0.61 SD units) compared to control. They also had higher birth length though BLGA-z was similar between the two groups (*p* = 0.24, Table 4). Similarly, fetal growth centiles were higher in the n-3 (43.7 %ile) vs. placebo (30.5 %ile) group (*p* = 0.02). There were no LGA children in either group. The percentage of SGA infants was 8% in the n-3 group and 17% in the placebo group, though the difference was not significant (*p* = 0.38).

### 3.6. Length of Gestation

Length of gestation was one week longer among women in the n-3 (40 (38.5, 40.1) weeks) vs. placebo (39 (38, 39.4) weeks) group (*p* = 0.02, Table 4). The percentage of preterm infants <37 weeks GA did not differ between the two groups (*p* = 0.3). There were no children born post-dates in this study.

### 3.7. Stratified Analyses

#### 3.7.1. Stratified Analyses by First Trimester BMI Category: Obese vs. Overweight

**Figure 2:**
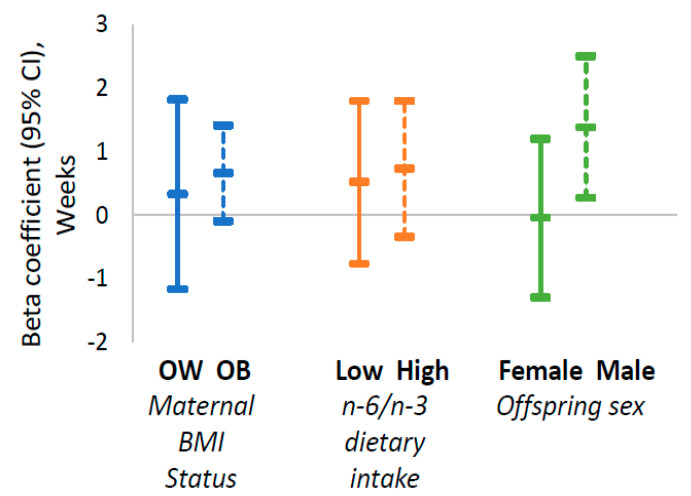
Effect of maternal n-3 PUFA supplementation on length of gestation by first trimester BMI status, pre-gravid n-6/n-3 dietary intake and offspring sex. Data shown are beta coefficients and their 95% CI estimated via linear regression with treatment group as exposure, using placebo as reference. In male offspring only, maternal supplementation during pregnancy with omega-3 was associated with longer gestation. Abbreviations: OW, overweight; OB, obese; BMI, Body mass index; n-6, Omega-6; n-3, Omega-3.

**Figure 3:**
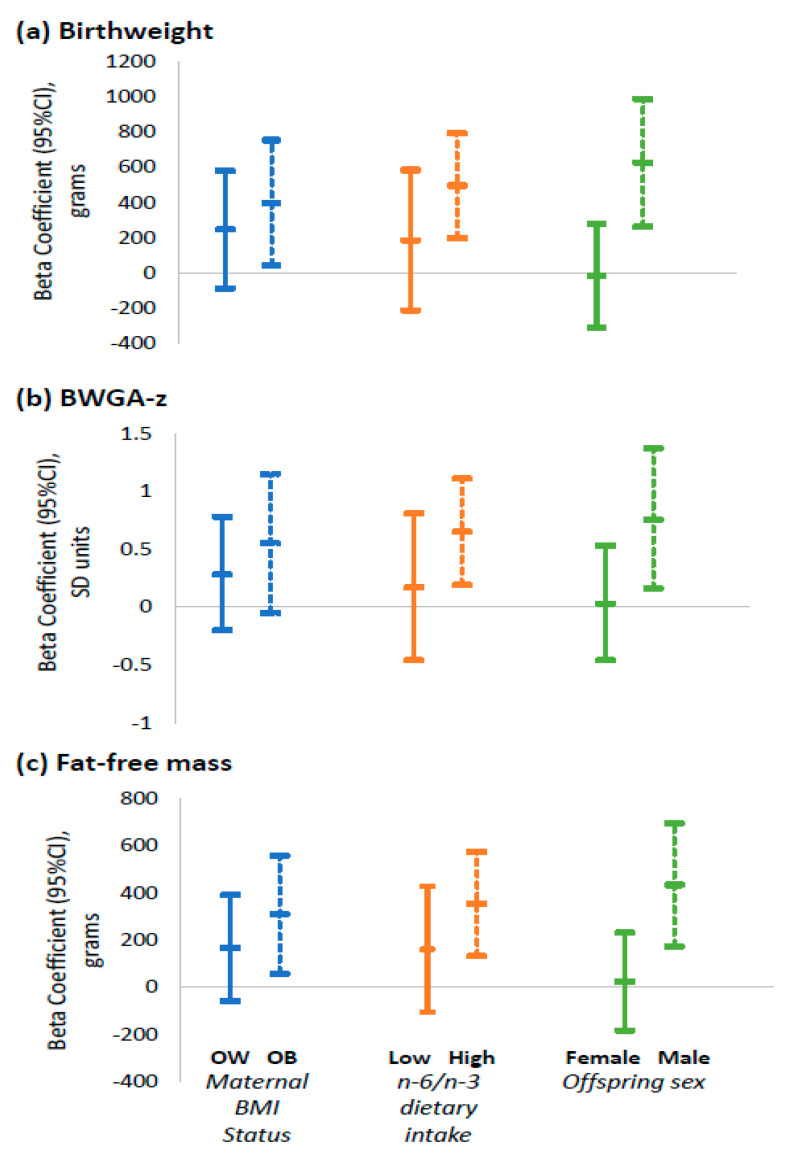
Effect of maternal n-3 PUFA supplementation on (**a**) Birthweight (**b**) BWGA-z score, and (**c**) Fat free mass estimated with the anthropometric equation, by first trimester BMI status, pre-gravid n-6/n-3 dietary intake and offspring sex. Data shown are beta coefficient and their 95% CI estimated via linear regression with treatment group as exposure, using placebo as reference. In women with obesity, supplementation was associated with higher birthweight and FFM. In women with high (above the median) pre-gravid dietary n-6/n-3 intake and in male offspring, supplementation was associated with higher birthweight, BWGA-z and FFM. Abbreviations: BWGA-z, Birthweight for gestational age *z*-score; OW, overweight; OB, obese; BMI, Body mass index; n-6, Omega-6; n-3, Omega-3.

Newborns of women with obesity supplemented with n-3 vs. placebo had higher BW (β: 399 g, CI: 44, 754) and FFM (Anthropometric FFM β: 308 g, CI: 59, 558) (Figure 2 and Figure 3). There were no differences in outcome measures in newborns of women with overweight supplemented with n-3 PUFA vs. placebo. However, P-interaction for maternal BMI category with treatment group was not significant (all P-int > 0.3). 

#### 3.7.2. Stratified Analyses by Baseline n-6/n-3 Dietary Intake

Newborns of women with a high (above median) early pregnancy n-6/n-3 dietary intake supplemented with n-3 vs. placebo had higher BW (β: 496 g, CI: 199, 792), BWGA-z (β: 0.65 SD units, CI: 0.19, 1.11) and FFM (Anthropometric FFM β: 352 g, CI: 131, 573) (Figure 2 and Figure 3). There were no differences in outcome measures in newborns of women with a low (below median) early pregnancy n-6/n-3 dietary intake supplemented with n-3 vs. placebo. However, P-interaction for maternal n-6/n-3 status with treatment group was not significant (all P-int > 0.2)

#### 3.7.3. Stratified Analyses by Infant Sex 

Supplementation with n-3 PUFA had a significantly greater effect on male compared to female neonates. The effect of n-3 supplementation during pregnancy on BW (the difference in effect of treatment in males vs. effect of treatment in females: β 639 g, CI: 188, 1090; P-interaction = 0.007) and the anthropometry-derived neonatal FFM (difference in β in males vs. females: 410 g, CI: 91, 729; P-int = 0.013) was stronger in male compared to female neonates (Figure 2 and Figure 3). The effect of n-3 supplementation on BWGA-z (difference in β in males vs. females: 0.73 SD units, CI: −0.02, 1.49; P-int = 0.06) and length of gestation (difference in β in males vs. females: 1.4 weeks, CI: −0.2, 3; P-int = 0.08).) was slightly stronger in males.

## 4. Discussion

In this randomized pilot study of a high-risk cohort of women with OWOB, we report for the first-time preliminary evidence of efficacy potential of n-3 supplementation. We showed that daily supplementation with 2 g of DHA and EPA starting early in pregnancy (~14 weeks) until delivery resulted in a 7-day longer length of gestation, 343 g higher birthweight, and 0.44 SD unit higher BWGA-z score. Interestingly, the higher fetal growth was not the result of higher adiposity, but instead reflected a higher fat-free mass accrual.

### 4.1. Neonatal Body Composition

Previous studies have suggested that infant adiposity at birth rather than BW is more sensitive to maternal dysmetabolism; therefore, evaluating body composition might provide better insight into maternal-fetal programming mechanisms. Here, we showed that offspring of women with OWOB supplemented with n-3 PUFA have higher FFM at birth, where FFM represents the combination of muscle mass, water and bone mineral content (BMC). Our findings are consistent with prior RCTs of n-3 supplementation in pregnant women which showed that higher maternal DHA status was related to higher offspring FFM at birth and early childhood without a concomitant change in FM or BF% [14,27,44]. However, results from RCTs on the role of perinatal n-3 supplementation on offspring body composition have been mixed, likely due to differences in methodologies, as well as the use of indirect growth variables such as waist circumference or absolute BMI [45] to assess adiposity. These measures provide less insight into true adiposity or obesity risk and might lead to inconsistent results about the effects of n-3 supplementation on adiposity accrual. 

Vinding [44] et al. showed in a recent study which evaluated children of women who participated in the COPSAC trial, that although fish oil supplementation in pregnancy was associated with higher BMI and waist circumference throughout the first 6 years of life, the risk of obesity at 6 years was not increased. Furthermore, similar to our findings, although children in the COPSAC trial had higher BMI, maternal n-3 PUFA supplementation led to significantly higher FFM including BMC, but not significantly higher FM and BF%. Our study demonstrates that reliance only on birthweight would have resulted in an interpretation that n-3 supplementation in women with OWOB results in larger babies assuming greater adiposity, while in fact this was not the case. This finding therefore highlights the importance of designing newborn outcome trials with specific measures of lean mass and adiposity. However, additional research is needed to understand the role of n-3 supplementation in OWOB pregnancies on offspring lean and fat mass accrual, and their impact on long-term obesity risk and cardiometabolic health.

The mechanisms via which n-3 PUFA might influence FFM accrual are unclear. Animal studies suggest that n-3 PUFA can promote calcium absorption and osteoblast formation which could increase bone density, growth and elongation [46,47]. Additionally, the insulin-sensitizing effects of n-3 PUFA may lead to increased activation of the placental mTOR signaling pathway, key regulator of placental amino acids transfer and protein synthesis [48]. Finally, maternal obesity is a state of chronic low-grade inflammation, and the anti-inflammatory effects of n-3 PUFA during pregnancy may enhance the commitment of mesenchymal stem cells in fetal muscle towards myogenesis [49]. However, further studies are needed to elucidate the role of n-3 PUFA in biological and molecular mechanisms that modulate fetal body composition in human pregnancies.

Several observational studies have shown associations between higher n-3 status in pregnancy or lactation and lower offspring adiposity in childhood [14,16,50]. We did not find that n-3 supplementation led to decreased newborn adiposity despite the observed reduction in placental inflammation and lipid storage and in plasma n-6/n-3 ratio, which have been shown to correlate with adipogenesis [51]. Our null findings with adiposity measures may be due to several factors. First, since this was a pilot study, we were not powered to detect significant effects in adiposity measures. We also previously showed that insulin sensitivity before conception (vs. during gestation) had the strongest correlation with newborn FM [52]. However, women in our trial began n-3 supplementation in the beginning of the second trimester and as a result we were not able to affect their pre-gravid markers of insulin sensivity. Furthermore, we and others have also shown that first trimester BMI is a strong independent predictor of neonatal adiposity and childhood obesity particularly in women who are OWOB pre-pregnancy [8,53,54]. Pre-gravid obesity may program adipose tissue development very early in the first trimester, as well as changes in placental function and gene expression linked to offspring growth and lipid metabolism [55,56]. Together with our results, these findings suggest that dietary and lifestyle interventions in women with OWOB may need to be initiated prior to conception or in the first trimester to optimize the metabolic *in-utero* milieu even earlier in pregnancy. 

### 4.2. Birthweight and Fetal Growth

We found that BW was higher in the n-3 PUFA group, and that the higher BW was not attributable only to longer gestation as has been previously suggested [22]. These findings are in line with recent RCTs and systematic reviews showing that n-3 PUFA supplementation is associated with increased fetal growth and lower incidence of BW < 2500 g in women of all weights [13,21,23]. Our results show greater effect size compared to these studies which found changes of ~97 g and ~172 g after n-3 supplementation. These differences might be related to lower doses of n-3 supplements and the later timing of intervention used in these other trials compared with our pilot RCT. They might also relate to the ameliorating effects of n-3 PUFA specifically in the *in-utero* metabolic milieu of OWOB pregnancies as we have previously demonstrated in this cohort [35,36]. Supplementation with n-3 PUFA in this cohort reduced inflammation, which is associated with improved placental angiogenesis, therefore improved fetal growth. Furthermore, Lee [57] et al. showed that n-3 supplementation during pregnancy was associated with epigenetic modification in imprinted genes involved in fetal growth and that this effect was increased in women with OWOB only, suggesting that the impact of n-3 supplementation on fetal growth might be modified by pre-pregnancy or first trimester BMI status. Interestingly, a RCT of fish oil and/or probiotics supplementation to decrease the incidence of GDM in women with OWOB found no difference in birthweight or fetal growth [58]; however, the fish oil composition differ in that DHA, and not EPA, was the dominant fatty acid in their fish oil supplement. A higher ratio of EPA to DHA (as in our pilot trial) may be important in evaluating the effects of n-3 PUFA supplementation on pregnancy outcomes as this ratio may contribute to greater homeostasis in maternal glucose and lipid metabolism, with subsequent *in-utero* effects on offspring body composition [59]. It is noteworthy here that in our pilot study, we observed a significant change in EPA concentrations, and not in DHA, following supplementation likely due to our fish oil supplements containing a higher (1.5×) ratio of EPA relative DHA.

Makrides [20] et al. demonstrated increased number of children born very LGA (>97% %ile) in the ORIP trial though the authors argued that this effect may have been due to chance since other anthropometric measures did not differ as a result of n-3 supplementation. We did not detect differences in the number of newborns born small or large for gestational age in the current study. Newborns in this study however had improved fetal growth as the average birthweight approached the expected normal mean for sex and gestational age. Women with obesity are at increased risk of pregnancies complicated by intrauterine growth restriction (due to inflammation-mediated reduced placental vascularization and placental insufficiency) and macrosomia (due to increased placental nutrient transfer as a result of insulin resistance), which are both linked to important metabolic disorders later in life [60]. Given the anti-inflammatory and insulin sensitizing effects of n-3 PUFA, RCTs in these higher risk women are therefore needed to evaluate the role of n-3 PUFA in preventing intrauterine growth restriction and macrosomia.

### 4.3. Length of Gestation

Our findings are in line with a Cochrane systematic review [13] that showed mean gestational length was greater in women who received n-3 PUFA, and a meta-analysis of 21 RCTs [24] that showed a 5.8-day increased gestation duration with n-3 supplementation in pregnancy. Our findings are unique in that our study was comprised specifically of women with OWOB who have an increased risk of preterm birth and a unique metabolism characterized by chronic low-grade inflammation, oxidative stress, and an imbalance in pro-inflammatory n-6 vs. anti-inflammatory n-3 PUFA. Together, these factors are thought to play a role in the process of labor initiation [61]. Christian [62] et al. has shown an increased risk for preterm birth (PTB) in the context of suboptimal n-6 to n-3 levels in African Americans (vs. Caucasians), which made up more than a third of our cohort. Although we were underpowered to assess for differences in PTB rates, Min [63] et al. demonstrated reduction in PTB in a fish oil supplementation trial of pregnant women with type 2 diabetes, and these women have characteristic metabolic dysregulation that is similar to women with obesity [64]. Furthermore, a secondary analysis of the ORIP trial showed that n-3 supplementation among women with a low total n-3 PUFA (≤4.1% of total fatty acids in whole blood) substantially reduced the risk for early preterm birth (<34 weeks) by 77% compared to control [29]. Results of the ORIP trial are consistent with our findings since women with OWOB in our cohort had a median total n-3 PUFA baseline concentration of 4.1%. All together, these studies suggest n-3 PUFA supplementation may play a role in the reduction of PTB in pregnancies complicated by low n-3 PUFA status and greater metabolic dysregulation and chronic inflammation. Therefore, well-powered clinical trials of n-3 supplementation specifically in women with OWOB, who generally have low n-3 relative to n-6 PUFA, and a high burden of PTB, are needed to determine the effects on their risk for preterm births.

### 4.4. Stratified Analyses

Results of our stratified analyses showed that the effects of n-3 supplementation were only evident in women with obesity or with a high dietary n-6/n-3 ratio. These findings are consistent with prior RCTs which showed that longer gestation and reduced risk for preterm birth as a result of n-3 supplementation were most evident among women with low n-3 PUFA concentration and with low dietary n-3 intake [28,29]. Pre-clinical studies of diet-induced maternal obesity also provide evidence of the biological plausibility of an effect of n-3 supplementation in these higher risk cohorts [12]. All together, these findings suggest that n-3 supplementation may be beneficial specifically in maternal-child dyads with overall lower diet quality and with the highest metabolic dysfunction. However, supplementing women who are already replete in omega-3 PUFA with additional omega-3 may increase risk for early preterm birth [29].

We also found significant interactions between n-3 supplementation and offspring sex whereby n-3 supplementation had stronger effects in males vs. females. This is contrary to previous studies that have not found significant sex-dependent effects in the setting of n-3 supplementation [21,27,44]. Animal studies, however, suggest that there are sex-specific placental adaptations to different pregnancy complications and that fetal vulnerability to *in-utero* conditions may be amplified in the male fetus [65]. Rodent studies suggest that male, rather than female, placentae are generally more susceptible to the effects of maternal overnutrition and inflammation [66]. Although the biological mechanisms contributing to this sexual dimorphism have not been fully elucidated, it has been postulated that perinatal androgens exposure and epigenetics may explain these differences. These findings suggest perhaps greater susceptibility to maternal nutritional and lifestyle interventions in male compared to female offspring. Indeed, a study by Carter et al. showed that maternal perinatal exercise, which improved maternal insulin sensivity, lead to increased percent lean mass in males but not females offspring [67].

### 4.5. Strengths and Limitations

Our findings must be interpreted within the context of study limitations. As a pilot study, it was not intended to be adequately powered to show significant differences between groups in our outcomes of interest. However, the current pilot study’s sample size was optimal in order to inform a future large RCT with 80% power to detect small-to-moderate effect size in fetal adiposity [68]. Although our significant results may have been due to chance, these results were nonetheless consistent with published literature. Furthermore, post-hoc power analyses revealed that a sample size of 48 maternal-infant dyads provided ~80% power to detect a 10% increase in birthweight and 2% increase in gestational age though we would have needed a sample size of 384 dyads to detect a 10% reduction in % body fat based on the current sample means. Additionally, the recruitment and retention rates suggest the need for further consideration given the known difficulties in recruitment in this population. The main barriers to compliance during the run-in, and to retention, which was 68% in this study, were participants’ difficulties with taking the 4 capsules/day, resulting in dropouts. Additional barriers included participant burden due to several metabolic studies in the study protocol, which included an oral glucose tolerance test, laboratory work, and body composition and resting energy expenditure evaluation; participants felt this was too much of a time commitment, resulting in drop-outs following randomization. Future studies should consider the use of more concentrated capsules, and more regular phone calls and support for participants in between research visits, as well as reduce the burden associated with research visits.

Despite these limitations, our study has several strengths. This is the first pilot study of n-3 supplementation specifically in a high-risk cohort of pregnant women with OWOB investigating newborn body composition, fetal growth and length of gestation. The limitation of supplementation studies is that usually the dietary fat content is not controlled which can lead to confounding. Here we assessed dietary intake in both treatment groups and showed that both groups’ diets were similar in the year leading to pregnancy as well during pregnancy. Therefore, the observed differences were likely not due to differences in dietary n-3 PUFA intake or to changes in their diet throughout pregnancy. We evaluated the response to n-3 supplementation by measuring maternal n-3 and n-6 plasma concentrations during the study. The concentrations of DHA+EPA was 36% higher in treatment vs. placebo at V2 which suggest good compliance to treatment assignment. We also started supplementation earlier (~14 weeks) than previous studies which is a strength given the role of the *in-utero* milieu early in pregnancy in metabolic programming. We used validated measures of body composition and evaluated the effect of n-3 supplementation specifically on FFM and FM, which have different effects on health. Finally, our cohort was racially diverse increasing the generalizability of our findings to more vulnerable populations.

## 5. Conclusions

Pre-pregnancy obesity is the strongest independent risk factor for child obesity and complications related to increased risk for preterm births. Women with pre-pregnancy obesity also have a low n-3 relative to n-6 PUFA status, which has been associated with preterm birth. This pilot study provides preliminary evidence that supplementation with n-3 PUFA during pregnancy in women with OWOB for ~25 weeks may increase fetal fat-free mass accrual, improve fetal growth, and increase length of gestation. Improving the n-3 status in pregnancy may have value as a prophylactic intervention in some women, particularly those with high-risk pregnancies such as women with obesity. Larger adequately powered trials of n-3 supplementation or dietary intervention specifically in women with OWOB starting before conception or early in pregnancy should be conducted to confirm these findings and explore the long-term impact on offspring adiposity and cardiometabolic health.

## Figures and Tables

**Table 1:** Participants’ Characteristics.

	Total		Placebo		Omega-3		*p* ^†^
	*n* = 48		*n* = 24		*n* = 24		
*Maternal Characteristics*	*Mean or n*	*SD or % or IQR*	*Mean or n* *or Median*	*SD or % or IQR*	*Mean or n*	*SD or % or IQR*	
	*or Median*				*or Median*		
Age (years)	26.9	5	27.1	4.8	26.7	5.2	0.89
BMI (kg/m^2^)	30.2	28.2, 35.4	29.5	27.2, 33	32.2	28.6, 36.4	0.24
BMI category [n (%)]							0.39
. Overweight	24	50	13	54	10	42	
. Obese	24	50	11	46	14	58	
Gestational weight gain (kg)	9.3	5.9	8.9	5.1	9.7	6.7	0.62
Race [n (%)]							0.21
. Caucasians	21	44	11	46	10	42	
. African American	17	35	6	25	11	46	
. Others	10	21	7	29	3	12	
Nulliparous [n (%)]							0.51
. Yes	12	25	5	21	7	29	
. No	36	75	19	79	17	71	
GA at recruitment (weeks)	14.8	13, 15.6	14.9	13.2, 15.8	14.6	12.8, 15.4	0.5
GDM [n (%)]							0.16
. Yes	5	10	4	17	1	4	
. No	43	90	20	83	23	96	

Abbreviations: n, number; SD, Standard deviation; IQR, Interquartile range; BMI, Body mass index; GA, Gestational age; GDM, Gestational diabetes. ^†^ *p*-values from *t*-test or Wilcoxon rank-sum test for continuous variables or chi-square test for categorical variables comparing treatment groups.

**Table 2:** Maternal dietary intake by treatment group.

	Placebo ^a^ (*n* = 23)				*p* ^†^	Omega-3 (*n* = 24)				*p* ^†^	*p* ^‡^	*p* ^§^
	Visit 1		Visit 2			Visit 1		Visit 2				
	*Median*	*IQR*	*Median*	*IQR*		*Median*	*IQR*	*Median*	*IQR*			
Calories (kcal/d)	2098	1423, 2900	2183	1103, 3259	0.44	1899	1648, 2564	1871	1615, 2420	0.84	0.67	0.78
Carbohydrates (g/day)	287	191, 415	280	150, 472	0.45	265	191, 356	260	202, 359	0.51	0.77	0.87
Sucrose (g/day)	54	34, 89	44	29, 81	0.35	53	33, 88	45	31, 82	0.28	0.81	0.93
Total sugars (g/day)	156	86, 253	145	68, 223	0.33	136	91, 198	128	94, 190	0.51	0.58	0.97
Total proteins (g/day)	84	51, 122	86	47, 144	0.93	72	60, 95	84	62, 110	0.24	0.58	0.83
Total fats (g/day)	77	54, 112	70	41, 119	0.38	72	53, 85	69	58, 81	0.69	0.59	0.9
ALA (n-3) (g/day)	1.3	0.8, 1.6	1.4	0.8, 1.9	0.92	1.1	0.9, 1.6	1.2	0.8, 1.4	0.51	1	0.25
LA (n-6) (g/day)	12	8, 15	13	8, 20	0.9	12	10, 16	13	8, 17	0.42	0.92	0.81
Total n-3 (g/day)	1.3	1.0, 1.8	1.6	0.8, 2.2	0.69	1.2	1, 1.6	1.4	0.9, 1.6	0.7	0.79	0.3
Total n-6 (g/day)	11	8, 17	13	8, 20	0.83	12	9, 16	13	8, 16	0.36	0.94	0.5
n-6/n-3 ratio (units/day)	9	8, 10	9	8, 10	0.65	9	8, 11	9	8, 10	0.44	0.75	0.8
Total PUFA (g/day)	14	10, 18	15	9, 23	0.95	14	11, 18	14	10, 18	0.27	0.93	0.58
Total SFA (g/day)	25	14, 38	24	14, 39	0.33	24	17, 29	24	18, 32	1	0.47	0.77

Abbreviations: n, number; IQR, Interquartile range; ALA, Alpha-linolenic acid; LA, Linoleic acid; n-3, Omega-3; n-6, Omega-6; PUFA, Polyunsaturated fatty acids; SFA, Saturated fatty acids. ^a^ No food frequency questionnaire data available for n = 1 participant in placebo group. ^†^ *p*-values from Wilcoxon signed-rank test comparing visit 1 vs. visit 2 for each group. ^‡^ *p*-values from Wilcoxon ranksum test comparing placebo vs. control at Visit 1. ^§^ *p*-values from Wilcoxon ranksum test comparing placebo vs. control at Visit 2.

**Table 3:** Plasma fatty acids concentration by treatment group.

	Placebo				*p* ^†^	Omega-3 ^a^				*p* ^†^	*p* ^‡^	*p* ^§^
	Visit 1		Visit 2			Visit 1		Visit 2				
	*n* = 24		*n* = 24			*n* = 23		*n* = 24				
	*Median*	*IQR*	*Median*	*IQR*		*Median*	*IQR*	*Median*	*IQR*			
Plasma EPA, (%mol)	0.2	0.2, 0.3	0.1	0.1, 0.2	0.002 *	0.2	0.2, 0.3	0.5	0.2, 1	0.002 *	0.28	<0.001 *
Plasma DHA (%mol)	2.8	2.4, 3.3	2.4	2.2, 2.8	<0.001 *	2.8	2.5, 3.3	2.9	2.2, 3.7	0.61	0.81	0.03 *
Plasma DHA+ EPA (% mol)	2.9	2.7, 3.5	2.5	2.3, 3	<0.001 *	3.1	2.7, 3.6	3.4	2.5, 4.4	0.08	0.83	0.01 *
Plasma total n-3 (%mol)	4.1	4, 4.5	3.5	3.3, 4	<0.001 *	4.1	3.8, 4.6	4.3	3.6, 5.5	0.12	0.85	0.007 *
Plasma total n-6 (%mol)	44	41.3, 45.9	40	37.7, 42.6	<0.001 *	42.1	39.2, 46.2	39.1	37.1, 42	<0.001 *	0.54	0.52
Plasma AA (%mol)	8.1	7.2, 9.3	6.4	5.5, 7.4	<0.001 *	8.3	6.8, 9.8	6	5, 6.6	<0.001 *	0.85	0.20
Plasma total n-6/n-3 ratio	10.6	9.6, 11.1	11.7	9.9, 12.3	0.01 *	10.2	9.4, 11.7	9.5	6.5, 11.8	0.06	0.78	0.02 *
Plasma AA/DHA+EPA ratio	2.6	2.4, 3.1	2.4	2.2, 2.7	0.008 *	2.7	2.1, 3.2	1.7	1.1, 2.3	<0.001 *	0.75	0.003 *
Plasma total SFA (%mol)	31.3	30.3, 32.6	32.9	31.7, 34.2	<0.001 *	31.7	30, 33.1	32.9	31.3, 34.5	0.003 *	0.8	0.87

Abbreviations: n, number; IQR, Interquartile range; EPA, Eicosapentaenoic acid; DHA, Docosahexaenoic acid; n-3, Omega-3; n-6, Omega-6; AA, Arachidonic acid; SFA, Saturated fatty acids. ^a^ No plasma available for n =1 participant in omega-3 group at visit 1. * Indicates statistically significant difference between groups. ^†^ *p*-values from Wilcoxon signed-rank test comparing visit 1 vs. visit 2 in placebo and omega-3 groups. ^‡^ *p*-values from Wilcoxon ranksum test comparing placebo vs. control at visit 1. ^§^ *p*-values from Wilcoxon ranksum test comparing placebo vs control at visit 2.

**Table 4:** Offspring body composition, fetal growth and length of gestation by treatment group.

	Placebo		Omega-3		*p ^†^*	*β (CI) ^a^*	
	*n* = 24		*n* = 24				
	*Mean or n* *or Median*	*SD or % or IQR*	*Mean or n* *or Median*	*SD or % or IQR*			
Anthropometry-derived body composition measures							
FM (g)	307	127	396	160	0.04 *	71 (−16, 158)	
FFM (g)	2628	250	2883	310	0.003 *	218 (49, 387) *	
Body fat (%)	10.1	3.5	11.7	3.8	0.16	1.2 (−1, 3.4)	
Anthropometrics						N/A	
Birthweight (g)	2935	356	3278	448	0.005 *		
Birth length (cm)	48.1	1.9	49.4	1.9	0.02 *		
Fetal growth						N/A	
BWGA z-score (SD units)	−0.61	0.61	−0.17	0.67	0.02 *		
BLGA z-score (SD units)	−0.56	0.62	−0.33	0.73	0.24		
Growth centile (percentile)	30.5	18.5	43.7	20.7	0.02 *		
Size for gestation					0.38		
AGA, 10–90 %ile [n %)]	20	83	22	92			
SGA, <10 %ile [n (%)]	4	17	2	8			
LGA, >90 %ile [n (%)]	0	0	0	0			
GA (weeks)	39	38, 39.4	40	38.5, 40.1	0.02 *	N/A	
Preterm delivery, GA < 37 weeks [n (%)]	3	13	1	4	0.30	N/A	

Abbreviations: n, number; *β*, Beta coefficient; CI, Confidence interval; SD, Standard deviation; IQR, Interquartile range; GA, Gestational age; BWGA-z, sex and GA-adjusted birthweight per Fenton growth standards; BLGA-z, sex and GA-adjusted birth length per Fenton growth standards; AGA, Appropriate for gestational age; %ile, percentile; SGA, Small for gestational age; LGA, Large for gestational age; FM, Fat mass; FFM, Fat free mass. ^†^ *p*-value from *t*-test or Wilcoxon rank-sum test or Chi-square test comparing treatment groups. ^a^ Sex-adjusted estimates for the difference between omega-3 vs. placebo (reference) in body composition measures from linear regression analyses. * Indicates statistically significant difference between groups.

## Data Availability

The data presented in this study are available on request from the corresponding author.

## Supplementary Materials

The following are available online at https://www.mdpi.com/2072-6643/13/2/578/s1, Table S1: Peapod-derived neonatal body composition by treatment group.
