# Supplementary material for: Effect of Omega-3 Supplementation in Pregnant Women with Obesity on Newborn Body Composition, Growth and Length of Gestation: A Randomized Controlled Pilot Study
Source: Nutrients. 2021 Feb 9;13(2):578. doi: 10.3390/nu13020578 (PMC7916127; doi:10.3390/nu13020578)
Supplement: Supplementary file 1 [file nutrients-13-00578-s001.pdf]

Table S1. Peapod-derived neonatal body composition by treatment group

	Placebo n = 20		Omega-3 n = 20		$P^{\dagger}$	β (CI) [a]
	Mean or n or Median	*SD or % or IQR*	*Mean or n or Median*	*SD or % or IQR*		
PeaPod measures[c]						
FM (g)	448	142	471	139	0.61	15 (-82, 112)
FFM (g)	2561	229	2807	328	0.009*	182 (-4, 369)
Body fat (%)	14.7	3.6	14.2	3.4	0.67	-0.42 (-2.8, 2)

Abbreviations: n, number; β, Beta coefficient; CI, Confidence interval; SD, Standard deviation; IQR, Interquartile range; FM, Fat mass; FFM, Fat free mass

†*P*-value from *t*-test or Wilcoxon rank-sum test or Chi-square test comparing treatment groups

[a] Beta coefficient (and 95% CI) for the difference between omega-3 vs. placebo (reference) in body composition measures obtained from linear regression analyses adjusted for sex

*Indicates statistically significant difference between groups
